# Effects of Different Afforestation Measures on Biological Soil Crust Properties and Microbial Communities in an Alpine Sandy Land

**DOI:** 10.3390/biology14111530

**Published:** 2025-10-31

**Authors:** Shaobo Du, Huichun Xie, Gaosen Zhang, Feng Qiao, Guigong Geng, Chongyi E

**Affiliations:** 1 College of Geographical Sciences, Qinghai Normal University, Xining 810008, China; wo827288809@163.com; 2 Qilian Mountain Southern Slope Forest Ecosystem Research Station, Huzhu 810500, China; qiaofnm@163.com; 3 Key Lab of Medicinal Animal and Plant Resources of Qinghai-Tibetan Plateau in Qinghai Province, Qinghai Normal University, Xining 810008, China; 4 College of Life Sciences, Qinghai Normal University, Xining 810008, China; 5 Key Laboratory of Extreme Environmental Microbial Resources and Engineering, Northwest Institute of Eco-Environment and Resources, Chinese Academy of Sciences, Lanzhou 730000, China; gaosenzhang@hotmail.com; 6 Qinghai Academy of Agriculture and Forestry Sciences, Qinghai University, Xining 810016, China; genggg-298@163.com

**Keywords:** desertification control measures, alpine sandy land, biological soil crust, physicochemical properties, enzymatic activity, bacterial community structure

## Abstract

**Simple Summary:**

Alpine sandy areas in the Gonghe Basin, which had been subjected to four afforestation measures, exhibited a gradual increase in water content, nutrient content, enzymatic activities, and bacterial community richness and diversity with succession from the bare sand stage to an algae crust and then to a moss crust. In particular, the above indicators for the two types of biological soil crusts had higher values at the *Populus simonii* plantation (YY) site than at other afforested sites. Effective phosphorus and organic matter were the primary environmental factors affecting bacterial community structure in algae and moss crusts, respectively, and the activities of four types of enzymes were significantly correlated with the relative abundance of most major bacterial phyla. The functional prediction results showed that YY preserved the balance of primary functions while offering precise support for the physiological characteristics and ecological needs of different crust types in the secondary functions. A comprehensive analysis of the indicators revealed that YY was more favorable for the development of biological soil crusts.

**Abstract:**

A good understanding of the effects of different afforestation measures in alpine sandy land on the physicochemical properties, enzymatic activities, and bacterial community structure of such crusts enables elucidation of the succession patterns of biological soil crusts and provides a theoretical basis for precise optimization of desertification control programs in alpine sandy land. In the present study, four afforestation measures—*Salix cheilophila*+ *Populus simonii* (WLYY00), *S. cheilophila* (WL), *P. simonii* (YY), and *Caragana korshinskii* (NT00) plantations—were adopted. The physicochemical properties and enzymatic activities of bare sand, algae crust, and moss crust in the four afforested sites were analyzed using Illumina high-throughput sequencing and PICRUSt2 functional prediction to investigate the bacterial community structure and function. Results indicated the following: (1) Water content, nutrient content, enzymatic activities, and bacterial community richness and diversity increased stepwise with succession from the bare sand stage to algae crust and to moss crust. The enhancement effect of YY on the above indicators and fine particle content was most prominent. (2) The primary environmental factors affecting bacterial community structure in algae and moss crusts were adequate phosphorus and organic matter, respectively, and the correlations between the activities of the four enzymes and the bacterial community structure are also quite close. (3) Functional prediction indicated that metabolism was the main primary function of biological soil crusts at the various sample sites. YY maintained the balance of primary functions and provided precise support for the physiological characteristics and ecological needs of different crust types in the secondary functions. In conclusion, among the four types of afforestation measures with a restoration period of 24 years, YY provided a greater advantage in improving the nutrient content, bacterial community structure, and functional potentials of biological soil crusts. The results of this study can serve as a scientific reference for screening of afforestation measures and protecting and utilizing biological soil crusts during the ecological restoration of alpine sandy lands in the present study area and other regions.

## 1. Introduction

Desertification is a severe ecological and environmental problem worldwide. It leads to a series of land degradation phenomena such as reduced soil water retention and productivity, resulting in the formation of large areas of sandy land [1,2]. Biological soil crusts (BSCs), formed by the cementation of algae, lichens, mosses, bacteria, and fungi to ground particles, are widely distributed on the surface layer of sandy land [3]. They are primarily divided into three successional stages: algae, lichen, and moss crusts. In particular, algae and moss crusts are the most common crust groups in sandy land due to their high biomass and wide coverage [4]. Algae can secrete high-molecular polymers such as polysaccharides to aggregate sand particles and form algae crusts on the soil surface, mainly including cyanobacteria, chlorophyta and diatoms. Among them, cyanobacteria play a key role in the formation and succession of crusts [5]. Moss crusts are organic complexes formed by the adhesion of rhizoids of Bryophyta plants to ground particles; they are in the advanced stage of the forward succession of BSCs. On sandy land, the main species that forms moss crusts is *Bryum argenteum* [6]. BSCs can serve various roles during the ecological restoration of sandy lands, including promoting vegetation succession, preventing wind erosion and stabilizing sand, retaining soil moisture, and enhancing soil nutrient content [7,8,9]. The strengths of these functions are closely related to the physicochemical properties and enzymatic activities of the crusts [10]. Relevant studies [11,12] have revealed significant differences in the physicochemical properties and enzymatic activities of BSCs subjected to different afforestation measures, as well as between BSCs at different developmental stages in identical environments. Therefore, elucidating the physicochemical properties and enzymatic activities of BSCs under different afforestation measures can provide a scientific basis for screening afforestation measures that effectively promote crust development and enhance its ecological function. This will be beneficial for the stable restoration and sustainable development of sandy land ecosystems. An understanding of changes in the physicochemical properties and enzymatic activities of BSCs across different developmental stages in the same environment can also shed light on the mechanisms of BSC succession and development. Bacteria are important in the formation and development of BSCs. Besides maintaining the structure and function of these crusts, bacteria can also drive ecosystem material cycling, influencing their physicochemical properties and enzymatic activities [13]. Therefore, an analysis of the composition and function of bacterial communities in BSCs enables an in-depth elucidation of the mechanisms of crust development and aids in assessing the restoration effect of afforestation measures through the analysis of differences in bacterial community function. This will contribute to the optimization of desertification control strategies.

With an extensive desertified land area of approximately 5573 km^2^, the Gonghe Basin is one of the most severely desertified areas in China [14]. The high altitude and low temperature of the region make it a typical alpine sandy land [15]. Large-scale afforestation measures have been implemented in the alpine sandy land of the Gonghe Basin since the 1960s to control the spread of desertification effectively [16]. Researchers have also systematically studied and evaluated the effectiveness of specific representative management measures in soil improvement [17,18]. However, these studies have merely assessed the soil improvement effects of different afforestation measures from the perspective of soil physicochemical properties, while the degree of development of BSCs, an important indicator, has been neglected. Zhang et al. [19] compared the differences in the physicochemical properties among various BSCs in areas subjected to three different afforestation measures. However, an in-depth investigation of the enzymatic activity, bacterial community structure, and function of the crusts was not reported. Therefore, our understanding of the response characteristics of BSCs to different vegetation types is limited, thereby restricting the precise optimization of ecological restoration strategies in alpine sandy regions. We selected alpine sandy areas of the Gonghe Basin that had been separately subjected to four representative afforestation measures in 2000 to achieve an extensive understanding of the effects of different afforestation measures on BSCs in alpine sandy land. Samples of bare sand without crust cover (0–2 cm soil layer), algae crust, and moss crust were collected. The physicochemical properties and enzymatic activities of the samples were measured, and bacterial community structure analysis and functional prediction were performed using high-throughput sequencing and PICRUSt2 functional prediction. Correlations among the physicochemical properties, enzymatic activities, and bacterial community structure of algae and moss crusts were subsequently determined. Our results revealed the response characteristics of physicochemical properties, enzymatic activities, and bacterial community structure and function of BSCs under different afforestation measures, elucidated the succession and development patterns of BSCs in alpine sandy land, and provided scientific support for the screening of optimal afforestation measures that can effectively promote the development of BSCs and strengthen their ecological functions. This can serve as a theoretical basis for promoting stable restoration and sustainable management of the alpine sandy land ecosystem in the Gonghe Basin.

## 2. Materials and Methods

### 2.1. Study Area

**Figure 1:**
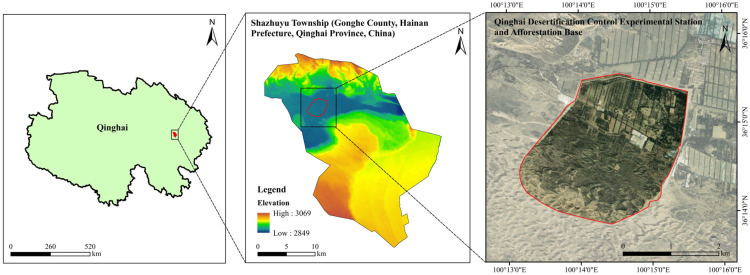
Overview of the study area.

The study area is located in a desertification control experimental station and afforestation base in Shazhuyu Township, Gonghe County, Hainan Prefecture, Qinghai Province, China (100°25′ E, 36°24′ N, Figure 1). Situated in the mid-western part of the Gonghe Basin, it belongs to the transition zone between alpine arid desert and semi-arid grassland [20]. The area is at an altitude of 2880 m and is characterized by an alpine climate, aridity, and intense radiation. Climatic characteristics include an annual precipitation of 264 mm, annual evaporation of 1528–1937 mm, average annual temperature of 2.4 °C, average annual wind speed of 2.7 m/s, and primarily northwesterly and westerly winds. The soil type is dominated by aeolian sandy soil, and BSCs are primarily algae and moss crusts, with the total coverage of other types of crusts being less than 1%.

### 2.2. Sample Site Selection and BSCs Sample Collection

Sample site selection and collection of BSC samples from the study area were performed in the second half of July 2024. Afforested sites on which the following four large-scale, representative afforestation measures had been implemented in 2000 were selected: *Salix cheilophila* + *Populus simonii* plantation (WLYY00), *S. cheilophila* plantation (WL), *P. simonii* plantation (YY), and *Caragana korshinskii* plantation (NT00) (Table 1). Prior to afforestation, all four sample sites were situated on mobile sand dunes on the alpine sandy land of the Gonghe Basin. Site conditions were generally consistent, with plant spacing being 1.5 m × 1.5 m. Afforestation was performed using pit sowing for NT00 and by planting seedlings in the remaining sites. Additionally, 3 large quadrats (50 m × 50 m) and 12 small quadrats (5 m × 5 m) were randomly established in the inter-dune area of each sample site. Based on the principles of random sampling and multi-point sampling, samples of bare sand (0–2 cm soil layer) without crust cover, algae crust, and moss crust were collected from each large quadrat. Samples of the same type were thoroughly mixed to form a single sample, and a total of 36 samples were obtained (3 large quadrats × 3 crust types × 4 sample sites). Each sample was divided into two parts: one part was used for the determination of physicochemical properties and enzymatic activities, and the other part was loaded into a 10 mL sterile centrifuge tube and stored in liquid nitrogen for subsequent bacterial sequencing. The coverage and thickness of crust samples within each small quadrat were recorded, and the average of the 12 small quadrats was calculated and used as the final value. During crust sample collection, a 9.0 cm diameter Petri dish was first used to delineate the sample collection area, and a sterile spatula was subsequently used to separate the crust layer from the underlying soil. The sampling tools were sterilized with 75% alcohol between sampling.

### 2.3. Physicochemical Properties and Enzymatic Activities

Table 2 shows the methods used for measuring physicochemical properties and enzymatic activities.

### 2.4. 16S rDNA Extraction and Sequencing

Extraction and PCR amplification of 16S rDNA were performed according to the methods described by Du et al. [27]. DNA purification kit: MagaBio Soil Genomic DNA Purification Kit (Thermo Fisher Scientific, Shanghai, China). The V3—V4 variable region of the soil bacterial 16S rRNA gene was PCR amplified using the universal primers 338F and 806R. The 16S rDNA library was constructed using the TruSeqTM DNA Sample Prep Kit (Illumina Corporation, San Diego, CA, USA), and sequencing was performed on the MiSeq PE300 platform. Upon the completion of sequencing, the MiSeq paired-end sequencing data were merged into one sequence using FLASH 1.2.11 software. After quality control in Fastp 0.19.6, the samples were differentiated using barcodes and primers at both ends to obtain valid sequences.

### 2.5. Data Processing and Analysis

Analysis of variance (ANOVA) and Duncan’s test for significant differences were performed in SPSS 27.0. Sequences were clustered into operational taxonomic units (OTUs) based on 97% similarity in UPARSE 7.0.1090, removing single sequences and chimeras during the clustering process, and compared to the Silva database to annotate species classification for each sequence. Alpha diversity indices (Coverage, Ace, Chao, Shannon, Simpson) were calculated in Mothur 1.30.2. Differences in the relative abundance of major bacterial phyla among different samples were assessed using the Kruskal–Wallis rank-sum test and one-way ANOVA. R software 3.3.1 was used to conduct inter-group difference tests with the alpha diversity index and to generate a principal coordinates analysis (PCoA) plot (calculate the sample distance matrix based on the distance algorithm of Bray–Curtis), soil bacterial community composition maps, correlation heatmaps, redundancy analysis (RDA) plots, and a Mantel test network heatmap.

PICRUSt2 functional prediction can be used to predict the functional information of bacterial communities in environmental samples, and to further understand some potential bacterial functional characteristics during environmental changes through functional composition and abundance. Functional prediction of the bacterial communities of samples was performed using the PICRUSt2 software based on the Kyoto Encyclopedia of Genes and Genomes (KEGG) database, and obtained the relative abundance of each function under the first-level and second-level function predictions

## 3. Results

### 3.1. Particle Composition of BSCs

**Figure 2:**
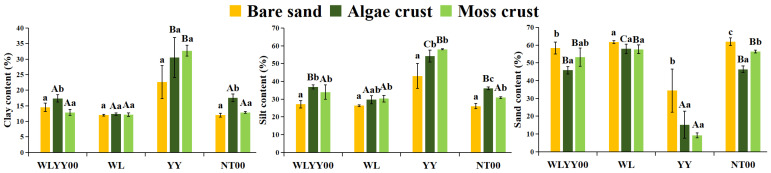
Particle composition. WLYY00: *Salix cheilophila* + *Populus simonii* plantation; WL: *S. cheilophila* plantation; YY: *P. simonii* plantation; NT00: *Caragana korshinskii* plantation. Different lowercase letters indicate significant differences in the indicators among bare sand, algae crust, and moss crust at the same site. Different uppercase letters indicate significant differences in the indicators of the same type of crust at different sites.

The particle compositions of bare sand, algae crust, and moss crust were mainly dominated by sand particles, accounting for more than 45%, at the WLYY00, WL, and NT00 sites. In contrast, these were dominated by silt particles, accounting for more than 42%, at the YY site (Figure 2). Further comparison of the particle composition characteristics of the four afforested sites revealed that algae and moss crusts had lower sand content and a higher content of fine particles (clay + silt particles) than bare sand at all sites. This reflected the enrichment effect of the BSCs on fine particulate matter. Notably, there were significant differences in the particle composition of the two types of soil crusts in different afforested sites. The algae crust had a higher fine particulate matter content at the WLYY00 and NT00 sites, the moss crust had a higher fine particulate matter content at the YY site, and the fine particulate matter contents of algae and moss crusts were comparable at the WL site. The clay and silt contents for the same type of crust were significantly higher and the sand content was significantly lower at the YY site than at all other sites. This result indicated that YY exerted the most significant promoting effect on the enrichment of fine particulate matter by BSCs.

### 3.2. Physicochemical Properties and Enzymatic Activities of BSCs

**Figure 3:**
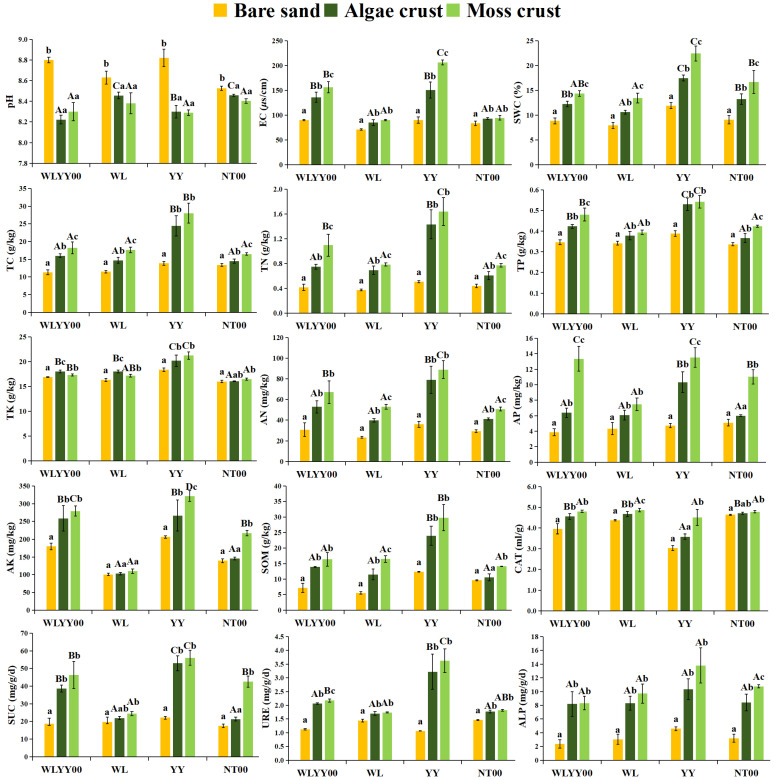
Physicochemical properties and enzymatic activities. WLYY00: *Salix cheilophila* + *Populus simonii* plantation; WL: *S. cheilophila* plantation; YY: *P. simonii* plantation; NT00: *Caragana korshinskii* plantation. Different lowercase letters indicate significant differences in the indicators among bare sand, algae crust, and moss crust at the same site. Different uppercase letters indicate significant differences in the indicators of the same type of crust at different sites.

Figure 3 shows that bare sand and two types of crusts in all four afforested sites were alkaline, with all pH values exceeding 8.22. The pH values of algae and moss crusts were significantly lower than that of bare sand, while there was no significant difference in pH between the two types of crusts. BSCs at the WLYY00 and YY sites had relatively lower pH values. Electrical conductivity (EC), soil water content (SWC), total carbon (TC), total nitrogen (TN), total phosphorus (TP), alkaline-dissolved nitrogen (AN), available phosphorus (AP), available potassium (AK), soil organic matter (SOM), catalase (CAT), sucrase (SUC), urease (URE), and alkaline phosphatase (ALP) values increased progressively from bare sand to algae crust to moss crust in each type of afforested site. At the WLYY00 and WL sites, the total potassium (TK) content of algae crust was significantly higher than that of moss crust, while moss crust had a significantly higher TK content than bare sand. At the YY and NT00 sites, TK content was highest in moss crust, but it was not significantly different from that of algae crust and was only significantly higher than that of bare sand. Among the four afforested sites, the YY site had higher contents of EC, SWC, and soil nutrients (TC, TN, TP, TK, AN, AP, AK, and SOM) than those of the same type of crust at the other sites. SUC, URE, and ALP activities of the same type of crust were also higher at the YY site than at the other sites, with only CAT activity being relatively low. These results indicated that YY was more conducive to promoting the improvement of physicochemical properties of BSCs on sandy land and also had a more pronounced effect on the enhancement of SUC, URE, and ALP activities, with only a relatively weak enhancement effect exerted on CAT activity.

### 3.3. Correlation Analysis of Physicochemical Properties and Enzymatic Activities

**Figure 4:**
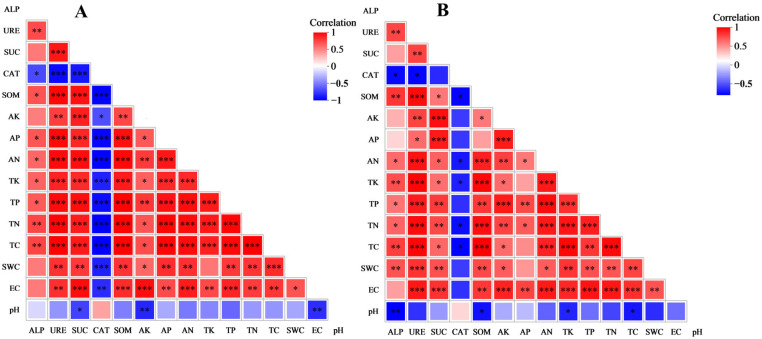
Correlations among physicochemical properties and enzymatic activities. (**A**,**B**) show the heatmaps of correlations between the physicochemical properties and enzymatic activities of algae crust and moss crust in different afforested sites, respectively; red denotes a positive correlation and blue denotes a negative correlation, with deeper colors indicating a higher degree of correlation. Asterisks within the color blocks indicate significance, *: *p* ≤ 0.05, **: *p* ≤ 0.01, ***: *p* ≤ 0.001.

The majority of physicochemical properties and enzymatic activities were significantly correlated in pairs in both algae and moss crusts (Figure 4). In both types of crusts, ALP was significantly positively correlated with TC, TN, TP, TK, AN, and URE, and significantly negatively correlated with CAT. The ALP of algae crust showed a significant positive correlation with AP, while the ALP of moss crust was significantly positively correlated with SWC and significantly negatively correlated with pH. URE was significantly correlated with all indicators—except pH, where it was significantly negatively correlated only with CAT—in both types of crusts. The SUC of algae crust had significant correlations with all physicochemical properties, CAT, and URE, being significantly negatively correlated with CAT and pH and significantly positively correlated with the other indicators. In moss crust, SUC had no significant correlations with CAT and pH, while its correlations with the rest of the indicators were in agreement with those of algae crust. In both types of crusts, CAT was significantly negatively correlated with TC, TN, TK, AN, and SOM. The CAT of algae crust was significantly negatively correlated with EC, SWC, TP, AP, and AK. In the algae crust, pH was significantly negatively correlated with AK and EC, exhibited no significant correlations with SWC and TK, and showed significant positive correlations with the remaining physicochemical indicators. In moss crust, pH was significantly negatively correlated with SOM, TK, and TC, and had no significant correlation with the other indicators; AP was significantly positively correlated with AK, EC, TN, TP, and AN, and had no significant correlations with other indicators; and the remaining indicators were significantly positively correlated with each other. The results described above revealed that the correlations between physicochemical properties and enzymatic activities in the four afforested sites were closer and more extensive in algae crust than in moss crust.

### 3.4. Structural Characteristics of Bacterial Communities in BSCs

#### 3.4.1. Statistical Analysis of OTUs

A total of 1,677,744 valid sequences were obtained from 36 BSC samples after bacterial sequencing. After clustering and transformation, 9898 OTUs belonging to 1 kingdom, 50 phyla, 141 classes, 322 orders, 540 families, and 1092 genera were obtained using classification and annotation. The bacterial community coverage index exceeded 0.977 in the various samples (Figure S1), indicating that the current sequencing volume was sufficient to cover the vast majority of species in the bacterial communities of bare sand, algae crust, and moss crust at the four afforested sites.

#### 3.4.2. Alpha Diversity Indices

**Figure 5:**
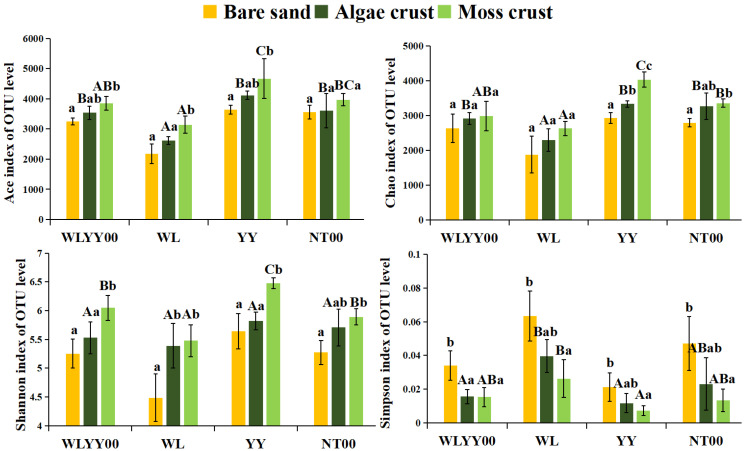
Alpha diversity indices of bacterial communities. WLYY00: *Salix cheilophila* + *Populus simonii* plantation; WL: *S. cheilophila* plantation; YY: *P. simonii* plantation; NT00: *Caragana korshinskii* plantation. Different lowercase letters indicate significant differences in the indices among bare sand, algae crust, and moss crust at the same site. Different uppercase letters indicate significant differences in the indices of the same type of crust at different sites.

The alpha diversity indices of bare sand and the two types of crusts in the four afforested sites were analyzed to assess the species richness and diversity levels of the bacterial communities (Figure 5). Specifically, the ACE and Chao indices were used to assess the richness of bacterial communities, while the Shannon and Simpson indices were used to characterize the diversity of bacterial communities. Figure 5 shows that the ACE, Chao, and Shannon indices gradually increased in all four afforested sites. In contrast, the Simpson index gradually decreased with succession from bare sand to algae crust and to moss crust. This indicated that both the richness and species diversity of the bacterial communities increased with the gradual maturation of crust development. Among the four afforested sites, the YY site had higher ACE, Chao, and Shannon indices and a lower Simpson index in the algae and moss crusts than the same crusts at the other sites. Therefore, it is evident that YY was more conducive for achieving an increase in richness and optimization of diversity of bacterial communities in the BSCs, as compared with the other three afforestation measures.

#### 3.4.3. Bacterial Community Composition

**Figure 6:**
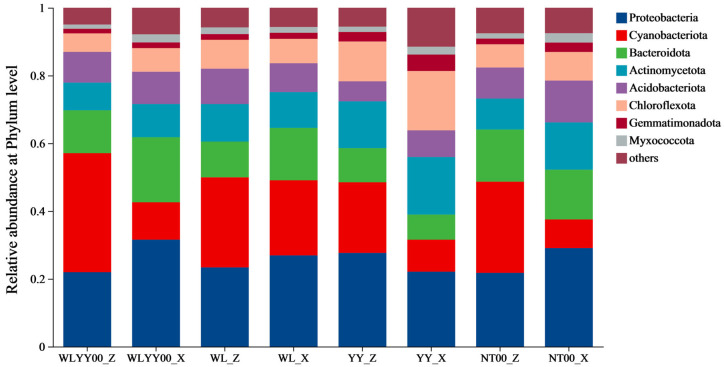
Bacterial community composition of BSCs. “Others” indicate all bacterial phyla with ranks higher than the 8th when sorted in descending order of relative abundance, sorted from high to low; WLYY00: *Salix cheilophila* + *Populus simonii* plantation; WL: *S. cheilophila* plantation; YY: *P. simonii* plantation; NT00: *Caragana korshinskii* plantation; _Z: algae crust; _X: moss crust.

Bacterial communities of algae crusts and moss crusts of the four afforested sites were mainly comprised of eight bacterial phyla (Figure 6). Proteobacteria, Cyanobacteriota, Bacteroidota, and Actinomycetota were the dominant phyla, with the relative abundances being 21.77–31.56%, 8.47–35.13%, 7.40–19.20%, and 8.17–16.99%, respectively. The relative abundances of Gemmatimonadota and Cyanobacteriota in the algae crust were lower and higher, respectively, than those of moss crust at all four afforested sites. Notably, the relative abundances of Cyanobacteriota in algae crust of the WLYY00, NT00, and YY sites were 3.19, 3.18, and 2.21 times that of the corresponding moss crusts, respectively. Other major bacterial phyla did not exhibit similar patterns of differences between algae and moss crusts.

#### 3.4.4. PCoA

**Figure 7:**
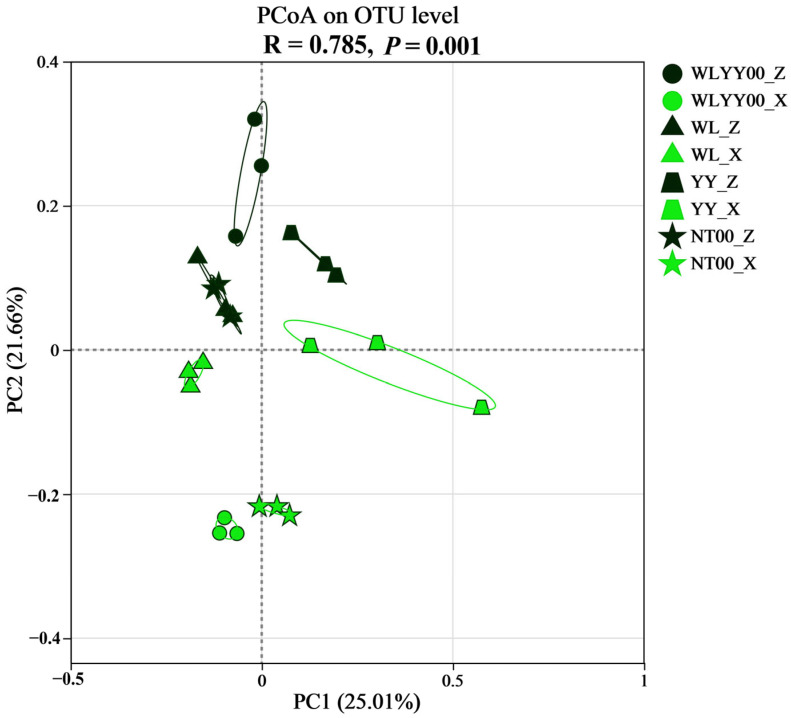
PCoA plot of bacterial communities. WLYY00: *Salix cheilophila* + *Populus simonii* plantation; WL: *S. cheilophila* plantation; YY: *P. simonii* plantation; NT00: *Caragana korshinskii* plantation; _Z: algae crust; _X: moss crust.

The PCoA plot shows the degree of similarity of bacterial community composition among different samples, with a smaller distance between sample points in the plot indicating higher similarity. PCoA performed in this study (*R* = 0.785, *p* = 0.001, Figure 7) revealed that the two types of crust samples each formed a relatively centralized distribution, suggesting higher similarity between bacterial community compositions of the same type of crust. The algae crust and moss crust sample points of the WL and YY sites were close to each other, which indicated that the bacterial community composition of the two types of crusts had greater similarity in these two afforested sites. However, at the WLYY00 and NT00 sites, the distributions of the two types of crusts were located further apart, demonstrating the presence of greater differences in bacterial community composition. This may be related to the selective shaping of bacterial communities in different crusts by unique microenvironmental conditions (e.g., moisture) in these two afforested sites, leading to clear differentiation of bacterial community composition between the two types of crusts. The bacterial community compositions of the same type of crust samples at the WLYY00 and NT00 sites had greater similarity, suggesting that the bacterial community structure of the same crust type possessed higher internal consistency under the influence of these two afforestation measures.

#### 3.4.5. Significance Testing of Intergroup Differences Among Major Bacterial Phyla

**Figure 8:**
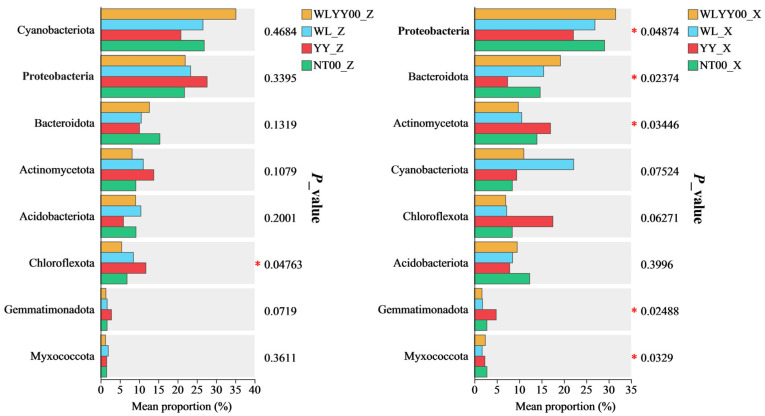
Significance testing of intergroup differences among major bacterial phyla. *: *p* ≤ 0.05; WLYY00: *Salix cheilophila* + *Populus simonii* plantation; WL: *S. cheilophila* plantation; YY: *P. simonii* plantation; NT00: *Caragana korshinskii* plantation; _Z: algae crust; _X: moss crust.

Significance testing of intergroup differences among eight major bacterial phyla in the algae and moss crusts of the four afforested sites was conducted (Figure 8). Results indicated that the relative abundances of Chloroflexota in algae crust and Proteobacteria, Bacteroidota, Actinomycetota, Gemmatimonadota, and Myxococcota in moss crust differed significantly among the four afforested sites. This indicated that on alpine sandy land, the effects of different afforestation measures with a restoration period of 24 years on the bacterial community of BSCs exhibited crust type specificity. The relative abundances of the major bacterial phyla in moss crust were more sensitive to responses to afforestation measures, and the structure of its major bacterial communities was susceptible to regulation by the afforestation measures. In contrast, the bacterial community structure of the algae crust was relatively stable.

### 3.5. Correlation Analysis of Physicochemical Properties, Enzymatic Activities, and Bacterial Community Structure

#### 3.5.1. Algae Crust

**Figure 9:**
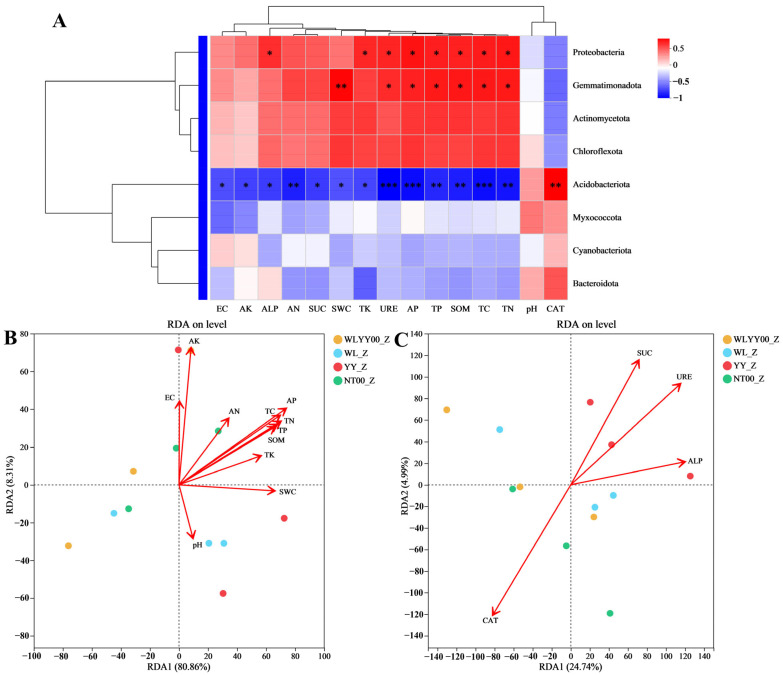
Correlations between environmental factors and bacterial community structure of algae crust. (**A**) Heat map of correlations of the physicochemical properties and enzymatic activities of algae crust with the relative abundances of major bacterial phyla. (**B**,**C**) RDA plots of bacterial community structure with the physicochemical properties and enzymatic activities of algae crust, respectively. WLYY00: *Salix cheilophila* + *Populus simonii* plantation; WL: *S. cheilophila* plantation; YY: *P. simonii* plantation; NT00: *Caragana korshinskii* plantation; _Z: algae crust; red denotes a positive correlation and blue denotes a negative correlation, with deeper colors indicating a higher degree of correlation. Asterisks within the color blocks indicate significance, *: *p* ≤ 0.05, **: *p* ≤ 0.01, ***: *p* ≤ 0.001.

Correlation analysis was performed on the physicochemical properties, enzymatic activities, and bacterial community structure of algae crust in the four afforested sites (Figure 9). Results revealed that most physicochemical properties and enzyme activity indicators are closely correlated with the bacterial community structure, but have no significant correlation with the relative abundance of multiple major bacterial phyla. Figure 9A shows that the relative abundances of Actinomycetota, Chloroflexota, Myxococcota, Cyanobacteriota, and Bacteroidota had no significant correlations with any physicochemical properties or enzymatic activities. The relative abundance of Acidobacteriota was not significantly correlated with pH but significantly correlated with all remaining indicators, with only CAT showing a significant positive correlation. Relative abundances of Proteobacteria and Gemmatimonadota were significantly positively correlated with URE, AP, TP, SOM, TC, and TN; the relative abundance of Proteobacteria was significantly positively correlated with ALP and TK; and the relative abundance of Gemmatimonadota was significantly positively correlated with SWC. From Figure 9B, it can be seen that AP was the most critical physicochemical factor affecting the community structure of algae crust bacteria in the four afforested sites of this study, followed by TC, TN, and TP. Figure 9C shows that the activities of the four enzymes are closely correlated with the community structure of algae crust, among which the correlation of URE is the most significant.

#### 3.5.2. Moss Crust

**Figure 10:**
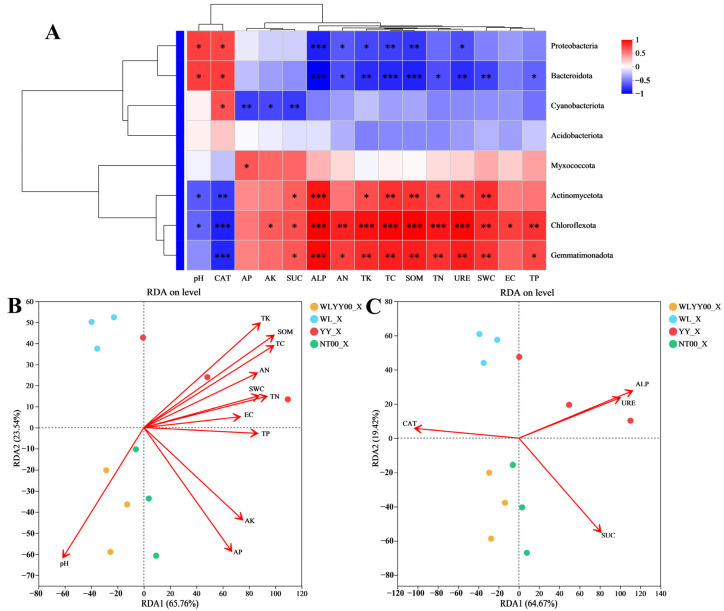
Correlations between environmental factors and bacterial community structure of moss crust. (**A**) shows the heat map of correlations of the physicochemical properties and enzymatic activities of moss crust with the relative abundances of major bacterial phyla; (**B**,**C**) show the RDA plots of bacterial community structure with the physicochemical properties and enzymatic activities of moss crust, respectively; WLYY00: *Salix cheilophila* + *Populus simonii* plantation; WL: *S. cheilophila* plantation; YY: *P. simonii* plantation; NT00: *Caragana korshinskii* plantation; _X: moss crust; red denotes a positive correlation and blue denotes a negative correlation, with deeper colors indicating a higher degree of correlation. Asterisks within the color blocks indicate significance, * *p* ≤ 0.05, ** 0.001 < *p* ≤ 0.01, *** *p* ≤ 0.001.

Correlation analysis was performed on the physicochemical properties, enzymatic activities, and bacterial community structure of moss crust in the four afforested sites (Figure 10). Results indicated that the physicochemical properties and enzyme activity indicators of moss crusts are closely correlated with the bacterial community structure, and also have a significant correlation with the relative abundance of most major bacterial phyla. This differed from the findings for algae crust described above. Figure 10A shows that the relative abundances of Proteobacteria and Bacteroidota were significantly positively correlated with pH and CAT, and significantly negatively correlated with ALP, AN, TK, TC, SOM, and URE, while the relative abundance of Bacteroidota was significantly negatively correlated with TN, SWC, and TP. The relative abundance of Cyanobacteriota was significantly positively correlated with CAT and significantly negatively correlated with AP, AK, and SUC. Relative abundances of Actinomycetota, Chloroflexota, and Gemmatimonadota were significantly negatively correlated with CAT and significantly positively correlated with SUC, ALP, TK, TC, SOM, TN, URE and SWC. The relative abundances of Actinomycetota and Chloroflexota were significantly negatively correlated with pH, the relative abundances of Chloroflexota and Gemmatimonadota were significantly positively correlated with AN and TP, and the relative abundance of Chloroflexota was significantly positively correlated with AK and EC alone. The relative abundance of Myxococcota was significantly correlated with AP only, with the correlation being positive, while the relative abundance of Acidobacteriota was not significantly correlated with any of the indicators. Figure 10B shows that SOM was the most critical physicochemical factor affecting the community structure of moss crust bacteria in the four afforested sites of this study, followed by TC and TN. From Figure 10C, it can be observed that the activities of the four enzymes are also closely related to the community structure of moss skinning bacteria, among which ALP is the most significant.

### 3.6. Functional Prediction of Bacterial Communities in BSCs of Different Afforested Sites

#### 3.6.1. Prediction of Primary Functions

**Figure 11:**
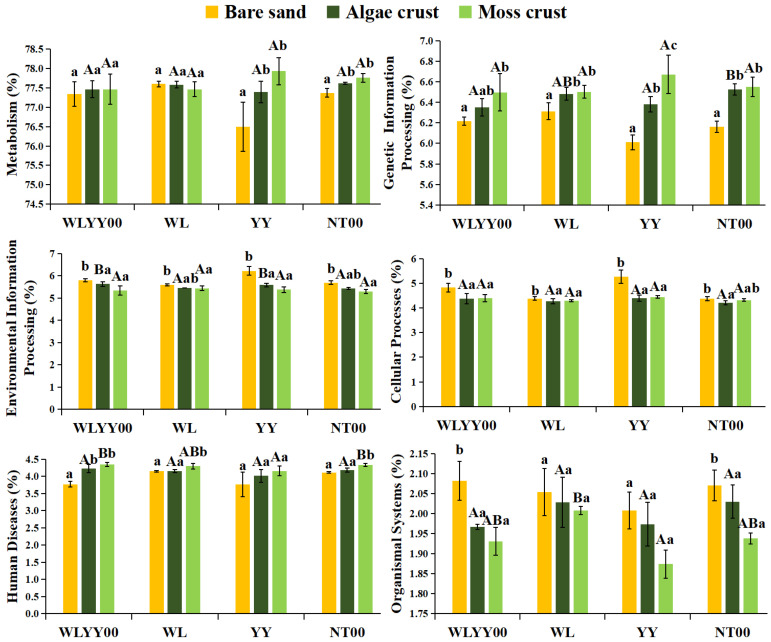
Relative abundances of primary functions of bacterial communities. Different uppercase letters indicate significant differences in the same type of crust across different afforested sites (*p* < 0.05), and different lowercase letters indicate significant differences among different types of crusts in the same afforested site (*p* < 0.05); WLYY00: *Salix cheilophila* + *Populus simonii* plantation; WL: *S. cheilophila* plantation; YY: *P. simonii* plantation; NT00: *Caragana korshinskii* plantation.

Functional prediction indicated that the bacterial communities of BSCs and bare sand in the four afforested sites possessed six primary functions. All relative abundances were greater than 1%, with metabolism having the highest relative abundance and accounting for more than 76% (Figure 11). The relative abundance of metabolism was not significantly different among bare sand, algae crust, and moss crust at the WLYY00 and WL sites. However, the relative abundances of metabolism in both crust types were significantly higher than that of bare sand at the YY and NT00 sites, with that of moss crust being higher than that of algae crust. With the succession from bare sand to algae crust and to moss crust in the four afforested sites, the relative abundance of genetic information processing and human diseases gradually increased. In contrast, the relative abundance of environmental information processing and organismal systems gradually decreased. The relative abundance of cellular processes in bare sand was significantly higher than that in algae and moss crusts, but did not differ significantly between the two types of crusts. Cross-site comparisons revealed no significant differences in the relative abundances of metabolism and cellular processes among the same type of crusts in the four afforestation sites. The relative abundances of genetic information processing and environmental information processing were not significantly different among moss crust of the various sites, and the relative abundances of human diseases and organismal systems were not significantly different among algae crust of the various sites. The relative abundance of genetic information processing in algae crust was considerably higher at the NT00 site than in other afforested sites. Relative abundances of environmental information processing in algae crust and human diseases in moss crust were the highest at the WLYY00 site. The relative abundance of organismal systems in the moss crust was the highest at the WL site, followed by that at the NT00 and WLYY00 sites. These results demonstrated that, except for YY, the other three afforestation measures were significantly targeted at enhancing the primary functions of bacterial communities in soil crusts. NT00 was more conducive to the exertion of the genetic information processing function in algae crust, WLYY00 provided optimal effects in promoting the environmental information processing function of algae crust and human disease function of moss crust, and WL was more favorable for the exertion of the organismal systems function of moss crust.

#### 3.6.2. Prediction of Secondary Functions

**Figure 12:**
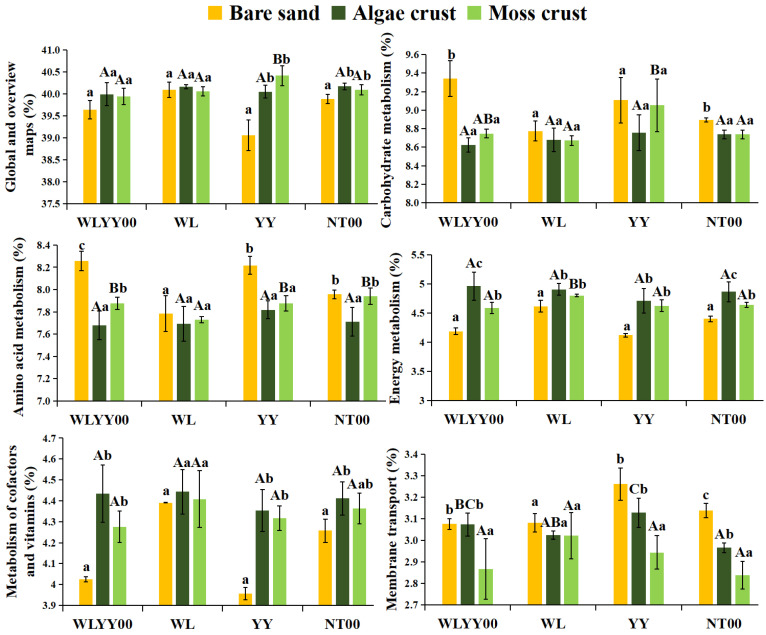
Relative abundances of secondary functions of bacterial communities. Different uppercase letters indicate significant differences in the same type of crust across different afforested sites (*p* < 0.05), and different lowercase letters indicate significant differences among different types of crusts in the same afforested site (*p* < 0.05); WLYY00: *Salix cheilophila* + *Populus simonii* plantation; WL: *S. cheilophila* plantation; YY: *P. simonii* plantation; NT00: *Caragana korshinskii* plantation.

Functional prediction indicated that the bacterial communities of BSCs and bare sand in the four afforestation sites possessed a total of 46 secondary functions. In the present study, only the top six bacterial community secondary functions in terms of relative abundance (accounting for more than 2.5% each) were retained. Global and overview maps had the highest relative abundance of approximately 40% (Figure 12). At the four afforested sites, the relative abundances of algae and moss crusts in global and overview maps were higher than that of bare sand, with the differences being statistically significant at the YY and NT00 sites. However, the relative abundance of this secondary function did not differ significantly between algae and moss crusts in the four afforested sites. The relative abundances of carbohydrate metabolism and amino acid metabolism in bare sand, algae crust, and moss crust of the four afforested sites exhibited the trend of bare sand > moss crust > algae crust, while the relative abundances of energy metabolism, metabolism of cofactors and vitamins, and membrane metabolism exhibited the trend of bare sand > algae crust > moss crust. This suggested that BSCs had a significant advantage in exerting the global and overview maps function, but showed a weaker performance than bare sand in other major secondary functions. The functional differences between the two crust types indicated that the algae crust was more likely to facilitate the exertion of functions such as energy and vitamin metabolism and membrane transport, while moss crust provided a greater advantage in functions such as carbohydrate and amino acid metabolism. Cross-site comparisons revealed that the relative abundance of membrane transport in algae crust was much higher at the YY site than at other sites, and the relative abundances of the remaining five secondary functions did not differ significantly among the four afforested sites. Among the moss crusts of the four sites, the relative abundances of global and overview maps and carbohydrate metabolism were highest at the YY site, the relative abundance of amino acid metabolism was highest at the WLYY00 site, and the relative abundances of energy metabolism, metabolism of cofactors and vitamins, and membrane transport were highest at the WL site. There was little variation in the relative abundances of multiple primary secondary functions of algae crust among the four afforested sites, which was similar to the results obtained from significance testing of intergroup differences among major bacterial phyla in algae crust. This again demonstrated that the structure of algae crust bacterial communities in alpine sandy land possessed high stability and was affected by the type of afforestation measure to a smaller extent. In contrast, the secondary functional characteristics of moss crust exhibited a clearer dependence on the type of afforestation measure. This suggested that functional differentiation was more significant in moss crust, and the expression of its related functions was more susceptible to regulation by afforestation measures. Therefore, targeted functional enhancement in moss crust may be achieved through the optimization of afforestation measures. When the functional advantages of the afforestation measures were examined, we observed that YY favored the promotion of the membrane transport function of algae crust and the global and overview maps and carbohydrate metabolism functions of moss crust. WLYY00 exceled in enhancing the amino acid metabolism function of moss crust, while WL improved the energy metabolism, metabolism of cofactors and vitamins, and membrane transport functions of moss crust.

## 4. Discussion

### 4.1. Characteristics of BSCs Particle Composition in Areas with Different Afforestation Measures

The alpine sandy land of the Gonghe Basin is prone to severe soil erosion. Wind and water erosion, the main forms of erosion in the region, directly affect soil particle composition. A lack of effective afforestation measures for soil protection will lead to continued erosion of the ground surface due to the lack of vegetation cover and difficulties in BSCs formation, resulting in a reduction in fine particle content in the soil [28,29,30]. In the present study, BSCs development in the study area achieved gradual maturation after 24 years of restoration under four different afforestation measures. The fine particle content of BSCs was higher than that of bare sand without crust cover. This indicated that BSCs reduced fine particle loss by wind transportation, which enabled the enrichment of fine particulate matter. However, at the WLYY00, WL, and NT00 sites, the particle composition of the two types of BSCs was still dominated by sand particles, which was similar to the results of a study by Du et al. [31] conducted in the Tengger Desert and a study by Cui et al. [12] conducted on the Erdos Plateau. This can be explained by the fact that the WLYY00, WL, and NT00 sites were located in the alpine sandy land of the Gonghe Basin, where the sand content in the original soil substrate was inherently high. The development of BSCs is primarily achieved by stabilizing the surface particles by cementation, making it challenging to fundamentally alter the basal particle composition of the regional soil [3]. At the YY site, the sand particle content of the two types of BSCs was significantly lower than that of the other afforested sites. The content of fine particles, such as clay and silt particles, was significantly higher than that of the other sites, with particle composition mainly dominated by silt particles. Such a result indicated that YY provided better effects in improving the particle composition of BSCs and promoting the enrichment of fine particulate matter (especially silt particles). YY also enabled effective retention and stabilization of fine particulate matter and promoted BSCs development towards finer granulation, which was more conducive to soil improvement. Differences in particle composition between the two types of crusts were relatively small. Significant differences were only observed at the NT00 site, where the algae crust had significantly higher fine particle and sand content than the moss crust. This suggests that the ability of algae crust to enrich fine particulate matter was superior to that of moss crust under the environmental conditions of the NT00 site.

### 4.2. Characteristics of Physicochemical Properties and Enzymatic Activities of BSCs in Different Afforested Sites

In sandy land ecosystems, afforestation measures serve as an important means of improving soil quality. Such measures can directly act on soil and regulate the basic soil environment by introducing litter and root exudates [32], while also indirectly improving soil quality by altering the physicochemical properties and enzymatic activities of BSCs [33,34]. Sandy lands are prone to imbalances in water-salt transport in soil due to scarce precipitation and high evaporation, a characteristic that makes them highly susceptible to soil salinization [35]. In the present study, the pH of algae and moss crusts was significantly lower than that of bare sand, and EC was significantly higher than that of bare sand. These differences in physicochemical properties were in agreement with the results obtained by Yan et al. [36] in the Tengger Desert. The heatmaps of correlations between physicochemical properties and enzymatic activities also demonstrated that pH was negatively correlated with EC. Therefore, BSCs can effectively alleviate further soil alkalization in sandy land, but may increase soil salinity to a certain extent through the retention or accumulation of salts in the crust layer. This represents a differential regulatory effect between the two key dimensions of salinization and alkalization. SWC was significantly higher in the algae and moss crusts than in bare sand, and the SWC of the same type of crust was significantly higher at the YY site than at other sites. Such a phenomenon can be explained by the fact that BSCs act as a special cover on land surfaces, stabilizing the soil. Their formation and development enable filling of degraded patches on land surfaces, thus influencing surface water circulation (e.g., infiltration and evaporation) and leading to improved water retention capacity in BSCs [37]. The high SWC level of BSCs at the YY site suggested the existence of microenvironments more suitable for crust development (e.g., vegetation cover and precipitation retention capacity) or higher developmental maturity of the crusts in this afforestation measure, which strengthened the soil water retention effect. We also found that moss crust had higher SWC and EC than algae crust. This demonstrated a superior water retention capacity and the ability to release more ion-containing organic or inorganic compounds in moss crust, thus further increasing the ionic concentration in the soil solution [38].

An examination of nutrient content and enzymatic activity characteristics in moss crust, algae crust, and bare sand revealed that, with the exception of TK content, which did not show consistent differences, all other nutrient and enzymatic activity indicators showed the trend of moss crust > algae crust > bare sand in the four afforested sites. This clearly reflected a trend of gradual enhancement in nutrient accumulation and enzymatic activity levels with an increase in the degree of BSCs development. This was consistent with the results obtained by Yang et al. [39] in a study on the differences in nutrient content between BSCs and bare sand in the Gurbantunggut Desert. Such a phenomenon is attributed to the presence of cryptogams within BSCs, which can fix carbon and nitrogen through photosynthesis and nitrogen fixation. This gives rise to more active carbon and nitrogen cycling processes compared with bare sand, which in turn promotes soil nutrient accumulation and enzymatic activities in BSCs [40,41,42]. Compared with algae crust, moss crust also possesses stronger photosynthetic and respiratory capacities and can secrete higher levels of enzymes through pseudo-roots and apoplasts, which contribute to higher nutrient content and enzymatic activities [11,40,43]. Our results also showed that the YY site had the highest nutrient content and ALP, URE, and SUC activities and the lowest CAT activity for the same type of crust across the four afforested sites. This demonstrated that YY was more conducive to promoting the accumulation of nutrients in BSCs and significantly enhancing the activities of most soil enzymes. Such a phenomenon can be ascribed to the use of *P. simonii* as the sand-fixing tree species at the YY measure. After 24 years of restoration, the root system of the trees had fully developed, with root length density being considerably higher than that of other sand-fixing plants. This enabled the secretion of higher levels of organic acids and organic substances through a larger root contact area, thereby enhancing soil nutrient content [44]. The weaker CAT activity suggested that the oxidative stress environment (e.g., reactive oxygen content) of BSCs at the YY site may differ from that of other sample sites, or that the antioxidant system may possess unique regulatory mechanisms.

### 4.3. Characteristics of Bacterial Community Structure and Functions of BSCs in Different Afforested Sites

Microorganisms are important components of BSCs. In particular, bacteria possess stronger adaptability in arid and semi-arid environments, and can promote soil material cycling and energy flow in sandy land ecosystems. Therefore, the environmental changes and functions of sandy land can be characterized by bacterial community structure [45,46]. In the present study, the dominant phyla of bacterial communities in BSCs of the study sites were Proteobacteria, Cyanobacteriota, Bacteroidota, and Actinomycetota. This finding is consistent with the community compositions of different types of BSCs in Mu Us sandy land reported by Li et al. [47]. Compared with the bacterial community composition of the surface soil of the alpine sandy land [18,27], the bacterial community composition of the BSCs had a higher relative abundance of Cyanobacteriota, which served as the dominant phylum. This is explained by the fact that Cyanobacteriota are the core builders of algae and moss crusts, and their secreted extracellular polysaccharides can provide a stable matrix for the attachment of other bacteria to form the basic crust framework. They also photosynthesize, promoting the conversion of carbon, nitrogen, and phosphorus, and primarily account for the higher nutrient content of the BSCs compared with that of bare sand [48]. Bacterial community richness and diversity levels of bare sand, algae crust, and moss crust followed the trend of sand < algae crust < moss crust in all afforested sites. Such a phenomenon can be explained as follows: BSCs in all four afforested sites had not yet fully developed. Therefore, nutrient saturation had not yet been reached, and competition among bacterial communities had not yet intensified. With a higher nutrient content, there was a greater abundance of resources, such as carbon, nitrogen, and phosphorus, available to bacteria, and a larger number of supporting bacterial communities. This contributed to higher community richness and diversity levels [49]. Significance testing of intergroup differences among major bacterial phyla revealed that different afforestation measures exerted more significant effects on the structure of major bacterial phyla of the bacterial community of moss crust. This was attributed to the significant improvement in soil physicochemical properties resulting from the different measures, combined with the greater susceptibility of the bacterial community structure of moss crust to the influence of soil environmental factors [50]. Bacterial community richness and diversity levels of the same type of biological crust were the highest at the YY site, due to the higher conduciveness of YY to improving the microhabitat conditions of biological crusts (e.g., enhancing nutrient supply, optimizing water retention, or stabilizing the physical structure). As a result, YY provided a suitable environment for the survival and reproduction of more types of bacteria, thus promoting the development of bacterial communities towards higher richness and complexity. AP and SOM were the primary environmental factors affecting the bacterial community structure of algae crust and moss crust, respectively, and TC and TN were the second and third environmental factors influencing both types of crusts. This was similar to the results of a study conducted in the Tengger Desert [51]. Therefore, despite differences in the alpine sandy land environment between the study area of the present study and the Tengger Desert, commonalities exist in the key environmental factors affecting the bacterial community structure of BSCs. The fact that both TC and TN were the second and third environmental factors affecting the bacterial community structure of both types of crusts suggests that the basic nutrients carbon and nitrogen served an important and universal regulatory role in the construction and stabilization of algae crust and moss crust bacterial communities in alpine sandy land, and were core common nutrient factors shaping the bacterial community composition in different types of BSCs. The correlations between the activities of the four enzymes and the bacterial community structure are all relatively close. This was due to the involvement of these enzymes in the cycling process of key elements such as carbon, nitrogen, and phosphorus in soil, which directly regulated the form and content of nutrients available to microorganisms in the soil and thus affected the bacterial community structure [52].

Functional prediction of bacterial communities in the BSCs revealed a total of six primary functions at each site, with metabolism being the main function. This was consistent with the results of functional prediction of soil bacterial community structures in the Hulun Buir Sandy Area by Du et al. [53], indicating that the distribution characteristics of primary functions are similar in other types of soils [54,55]. It can be deduced that the metabolic function serves as the core in bacterial communities, supporting the basic processes of soil ecosystems, and is not readily influenced by regional differences. Among the various secondary functions, global and overview maps, a secondary function subordinate to the primary function of metabolism, was identified as the main function. This suggested the need for bacterial communities to rely on coordination and regulation by the global and overview maps function to utilize the metabolism function fully. Notably, YY, which exhibited the most optimal improvement effects towards particle composition, physicochemical properties, enzymatic activities, and bacterial community structure in BSCs, did not show specific enhancement advantages for any of the six primary functions. However, it demonstrated significant differences in the secondary functions. For instance, YY was more conducive to promoting the exertion of the membrane transport function in the bacterial community of algae crust and the global and overview maps and carbohydrate metabolism functions in the bacterial community of moss crust. Therefore, YY maintained the balance of primary functions in function regulation and precisely supported the secondary functions according to the physiological characteristics and ecological needs of different types of crusts.

The results of this study indicate that, in terms of the response characteristics of BSCs to different afforestation measures, it is recommended to give priority to the YY measure in high-cold sandy land. This study has three main limitations. First, the sampling was only conducted in late July 2024, lacking a repetitive design in the time dimension. This makes it difficult for the research results to fully represent the characteristics of different seasons, especially failing to reflect the potential impact of environmental factor changes during seasonal transitions on the research subjects, significantly restricting the generalization and application of the conclusions on a cross-seasonal scale. Second, the sampling design did not fully consider the heterogeneity of microhabitats, which may result in some sample point data failing to fully capture the characteristics of small-scale habitats. Thirdly, although the composite sample processing method reduces random errors to a certain extent and smooths out local extreme values, it may also mask the subtle variations within the sample points, compressing the variances within the sample points and failing to fully reflect the true characteristics of a single sub-sample. Based on this, future research can enhance temporal representativeness by increasing the sampling frequency across multiple seasons, refining the sampling plan to cover microhabitat heterogeneity, and conducting comparative analysis of single samples and composite samples to further clarify the impact of different sample processing methods on data variation, thereby providing more comprehensive and reliable support for related research conclusions.

## 5. Conclusions

Biological soil crusts of the various afforested sites enriched fine particle content, increased soil water content, and reduced pH, thus enhancing soil resistance to wind erosion, improving water retention, and inhibiting further soil alkalization. Nutrient content, enzymatic activities, and bacterial community richness and diversity exhibited a trend of increase from bare sand to algae crust and to moss crust. Among the four afforestation measures with a restoration period of 24 years, YY was the most effective at enhancing biological soil nutrient content and optimizing bacterial communities. AP and SOM were the primary environmental factors affecting the bacterial community structure of algae and moss crusts, respectively. TC and TN were the second and third environmental factors affecting both types of crusts, and all four measured enzymatic activities exerted significant effects on bacterial community structure. Metabolism and global and overview maps were the main primary function and secondary function of biological soil crusts in sandy land, respectively. YY, which exhibited the best effects in regulating biological soil crust development, did not show specific enhancement advantages for any of the six primary functions. However, it promoted the exertion of the membrane transport function in the bacterial community of the algae crust and the exertion of global and overview maps, as well as carbohydrate metabolism functions in the bacterial community of moss crust.

## 6. Patents

[1] Shaobo Du, Huichun Xie, Chongyi E., Tianyue Zhao, Shuang Ji, Zhiqiang Dong, Shaoxiong Zhang, Haokun Wu. A plant fixation device for desertification control in deserts. ZL202322873122.9, 26 July 2024.
[2] Shaobo Du, Huichun Xie, Chongyi E., Shujing Qi, Haokun Wu, Shuang Ji, Tianyue Zhao, Zhiqiang Dong. An invention relates to a portable spraying device for desert algae biological control of sand. ZL202421424816.2, 28 March 2025.

## Figures and Tables

**Table 1:** Basic information of sample sites.

Sample Site	Longitude	Latitude	Elevation (m)	Area (m^2^)	Coverage	Crust Type	Crust Thickness/mm	Crust Coverage/%
WLYY00	100°14′27.23” E	36°15′32.432” N	2828	133,333	67%	Bare sand	nd	31.33 ± 11.47
						Algae crust	7.12 ± 1.06	41.62 ± 14.38
						Moss crust	13.11 ± 2.11	27.04 ± 4.31
YY	100°13′259.1” E	36°15′32.713” N	2827	46,666	81%	Bare sand	nd	13.01 ± 2.74
						Algae crust	4.74 ± 0.37	22.73 ± 4.13
						Moss crust	9.22 ± 1.43	64.25 ± 11.74
WL	100°14′16.54” E	36°14′50.678” N	2830	40,000	64%	Bare sand	nd	31.12 ± 9.48
						Algae crust	5.16 ± 0.85	27.18 ± 3.04
						Moss crust	8.45 ± 1.39	41.71 ± 8.45
NT00	100°14′36.26” E	36°15′5.3167” N	2825	133,333	91%	Bare sand	nd	17.23 ± 7.51
						Algae crust	4.16 ± 0.77	21.08 ± 6.36
						Moss crust	8.68 ± 1.46	61.70 ± 14.54

Note: Mean ± standard error; nd = not determined; WLYY00: *Salix cheilophila* + *Populus simonii* plantation; WL: *S. cheilophila* plantation; YY: *P. simonii* plantation; NT00: *Caragana korshinskii* plantation.

**Table 2:** Methods for determining physicochemical properties and enzymatic activities.

Indicator	Method	Reference
Soil particle composition	Laser method	[21]
pH	Potentiometric method (water/soil ratio of 2.5:1)	[22]
Electrical conductivity (EC)	Conductometric method (water/soil ratio of 5:1)	[22]
Soil water content (SWC)	Drying method	[22]
Alkaline-dissolved nitrogen (AN)	Alkaline dissolution and diffusion method	[22]
Available phosphorus (AP)	Sodium bicarbonate leaching and molybdenum antimony colorimetric method	[22]
Total phosphorus (TP)	Sodium hydroxide fusion and molybdenum antimony colorimetric method	[22]
Soil organic matter (SOM)	Potassium dichromate–concentrated sulfuric acid external heating method	[22]
Total carbon (TC) and total nitrogen (TN)	Combustion method	[22]
Total potassium (TK) and available potassium (AK)	Flame photometry	[22]
Urease (URE)	Indophenolic acid colorimetric method	[23]
Alkaline phosphatase (ALP)	Disodium phenyl phosphate colorimetric method	[24]
Sucrase (SUC)	3,5-Dinitrosalicylic acid colorimetric method	[25]
Catalase (CAT)	Potassium permanganate titration method	[26]

Note: According to American standards, soil particles are categorized into three classes: clay (<0.002 mm in diameter), silt (0.002–0.05 mm), and sand (>0.05 mm).

## Data Availability

The original contributions presented in the study are included in the article/Supplementary Material, further inquiries can be directed to the corresponding authors.

## Supplementary Materials

The following supporting information can be downloaded at: https://www.mdpi.com/article/10.3390/biology14111530/s1, Figure S1: Coverage index of bacterial communities. WLYY00: *Salix cheilophila* + *Populus simonii* plantation; WL: *S. cheilophila* plantation; YY: *P. simonii plantation*; NT00: *Caragana korshinskii* plantation; _W: bare sand; _Z: algal crust; _X: moss crust.

## Abbreviations

The following abbreviations are used in this manuscript:

BSCs	Biological soil crusts
WLYY00	*Salix cheilophila*+ *Populus simonii* plantation
WL	*Salix cheilophila* plantation
YY	*Populus simonii* plantation
NT00	*Caragana korshinskii* plantation
