# Supplementary material for: Effects of Different Afforestation Measures on Biological Soil Crust Properties and Microbial Communities in an Alpine Sandy Land
Source: Biology (Basel). 2025 Oct 31;14(11):1530. doi: 10.3390/biology14111530 (PMC12649861; doi:10.3390/biology14111530)
Supplement: Supplementary file 1 [file biology-14-01530-s001.zip › biology-3933978-supplementary.pdf]

Effects of Different Afforestation Measures on Physicochemical Properties, Enzymatic Activities, and Bacterial Community Structure of Biological Soil Crusts in Alpine Sandy Land

Supplementary Materials

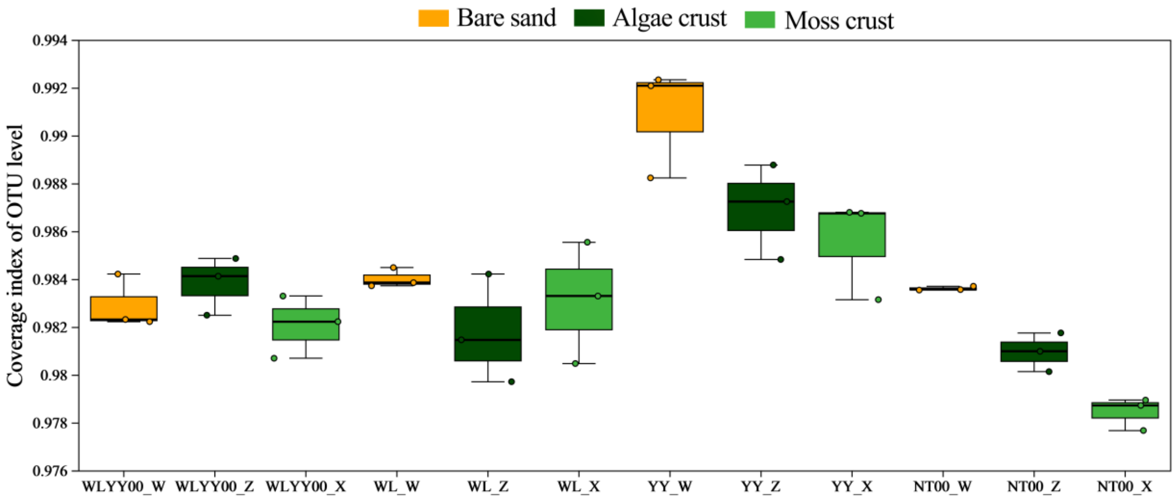

Figure S1. Coverage index of bacterial communities. WLYY00: *Salix cheilophila* + *Populus simonii* plantation; WL: *S. cheilophila* plantation; YY: *P. simonii plantation*; NT00: *Caragana korshinskii* plantation; _W: bare sand; _Z: algal crust; _X: moss crust.
